# Secondary Succession Altered the Diversity and Co-Occurrence Networks of the Soil Bacterial Communities in Tropical Lowland Rainforests

**DOI:** 10.3390/plants11101344

**Published:** 2022-05-19

**Authors:** Xuan Hu, Qi Shu, Wen Guo, Zean Shang, Lianghua Qi

**Affiliations:** 1 Key Laboratory of National Forestry and Grassland Administration/Beijing for Bamboo & Rattan Science and Technology, International Centre for Bamboo and Rattan, Beijing 100102, China; huxuan@icbr.ac.cn (X.H.); guowen@icbr.ac.cn (W.G.); 2 National Positioning and Monitoring Station for Ecosystem of Bamboo and Rattan, Sanya 572000, China; shuqi1005@caf.ac.cn; 3 Research Institute of Forestry, Chinese Academy of Forestry, Beijing 100089, China; 4 Shaanxi Academy of Forestry, Xi’an 710082, China; sza1001@sina.com

**Keywords:** secondary succession, tropical lowland rainforest, plant diversity, legumes niche overlap, bacterial community structure, network analysis

## Abstract

The characteristics of plant and soil bacterial communities in forest ecosystems have been reported, but our understanding of the relationship between plant communities and soil bacteria in different stages of secondary tropical rainforest succession is still poor. In June 2018, three different natural successional stages of tropical lowland rainforests, early (33 years), early-mid (60 years), and mid successional stage (73 years), in Hainan Island, China, were selected for this study. By conducting field investigation and 16S rRNA gene high-throughput sequencing, the composition and diversity of tree communities, the niche overlap of tree species with legumes among tree species, and the diversity and composition of soil bacterial communities and co-occurrence networks within communities across the successional stages were investigated. The results showed that plant richness and species diversity increased significantly during the secondary succession of tropical lowland rainforests. The order of positive correlations between nitrogen-fixing legumes and other species in plant communities was early-mid > mid > early successional stage. Soil nutrient content and soil bacterial richness were highest in the early-mid stages of succession, followed by mid and early stages of succession. Organic matter (OM), total nitrogen (TN), alkali nitrogen (AN), and available phosphorus (AP) had a stronger positive impact on soil bacterial communities. Co-occurrence network analysis showed that with the advancement of rainforests succession, the negative correlation between soil bacterial species decreased, and the community stability increased. Overall, as a result of tropical lowland rainforest secondary natural succession, the richness and diversity of plant communities increased, which altered the living conditions of nitrogen-fixing legumes and the soil properties, and the network complexity of soil bacterial communities increased with the rising of rainforest soil nutrient content.

## 1. Introduction

Secondary succession, also known as natural restoration of the vegetation, has been regarded as a low-cost and effective method and strategy of increasing forest cover, biodiversity, and soil functions [1,2]. There have already been some global findings on the dynamics of plant and soil microbial communities as they relate to forest succession, indicating that secondary forests may be able to restore species richness and composition to primary forest levels within decades to hundreds of years of succession [3], and that vegetation can alter the soil microbial community and structure by changing soil characteristics [4] and maintaining soil fertility, either directly or indirectly [5]. However, there are still some unanswered questions in these studies about what pattern changes in forest soil microbial interactions during natural succession progress, and whether there is a direct connection between the changes in microbial communities and the development of plant communities.

The composition, structure, diversity, and interspecies relationship of plant communities changes over time following forests disturbance as a result of both above and underground interactions [6,7]. Plant composition and community structure can affect small-scale environmental changes in forests. For example, different stand densities and canopy closures can affect the lighting conditions in the forest [8], and the type and thickness of litter can alter the surface temperature and humidity [9]. Plant root exudates and leaf litter decomposition can affect soil nutrient content and environmental conditions such as pH [10]; these changes of vegetation can have a significant impact on soil microbial communities [11], which may in turn feed back to these changes of vegetation and soil. A large number of studies have shown that legumes can have a certain impact on soil nitrogen content due to their special biological characteristics [12,13,14]. However, outside the agricultural system, the relationship between legumes and surrounding plant communities and the influence or interaction of legumes in forest ecosystem is poorly understood.

Bacteria are the most abundant part in the soil [15]; in the process of forest disturbance they are more sensitive than fungi [7] but have greater abilities in restoration [16]. Therefore, the succession dynamics of the soil bacterial community can better reflect the process of forest succession; at the same time, it can express the impact of aboveground plant community changes on it. In general, later stages of forest succession have higher plant diversity than earlier stages and higher plant diversity can import more nutrients to forest soil. At the same time, the forest soil in the later succession has a higher water retention capacity because of the continuous development of the plants’ root system [17], which may affect the growths of soil bacterial communities composition, structure, and diversity. With the succession of the forest, the stand canopy density continued to rise, and this had a significant impact on the light-sensitive bacterial abundance and community composition [18].

Tropical forests are one of the most significant biomes on the planet. Despite accounting for only 12% of the total land area, they carrying an estimated 50% of species and store 25% of plant biomass, serving as global warehouses for biodiversity and carbon [19]. Additionally, tropical forests provide vital ecosystem services such as staple food and fruit, timber and rattan products, erosion control, biodiversity conservation, and water regulation in addition to influencing climate [20,21]. Natural disasters and human activities such as logging, pasture conversion, and cash crop agriculture are putting tropical forests in jeopardy [22], leading to less than 50% of the world’s tropical forests remaining standing today [23]. With the lower altitude, tropical lowland rainforests are more susceptible to external human interference, which destroys forest vegetation communities, resulting in changes in the internal environment. The degeneration of tropical rainforests may have serious implications for biodiversity, climate normalization, and the well-being of human beings [24]. Throughout the history of the earth’s crust, Hainan Island, which has the largest area of island-type tropical rainforests and the greatest plant species diversity in China, has had repeated separations and reunions with the mainland. This region served as a biological sanctuary during the ice age, retaining a varied range of biological species due to its unusual geographical location [25], which therefore provides an ideal opportunity to examine the effect of secondary succession on the bacterial diversity, networks, tree community composition, diversity, and interspecies relationship of tropical lowland rainforest.

In this study, we selected three tropical lowland rainforests disturbed by deforestation, with different secondary succession stages (Table 1) in Hainan, China, namely early successional stage (33 years), early-mid successional stage (60 years), and mid successional stage (73 years). Field investigation and 16S rRNA gene high-throughput sequencing were conducted to investigate the composition and diversity of tree communities, the niche overlap of tree species with legume tree species, and the diversity and composition of soil bacterial communities, as well as network relationships within these communities. We hypothesized that in the process of natural rainforest secondary succession, the plant community’s diversity and interspecific relationships would affect soil properties, which in turn would affect the network complexity of soil bacterial communities. Our objective was to (1) investigate the patterns of change in soil properties, plant community composition and diversity, and soil bacterial community composition and diversity at three different rainforest successional stages, (2) explore whether there are special plant groups (such as legumes) that have a unique impact on rainforest environmental changes, (3) determine the response of soil bacterial community networks along the tropical lowland rainforest natural secondary succession.

## 2. Results

### 2.1. Plant Community Composition and Diversity

**Figure 1:**
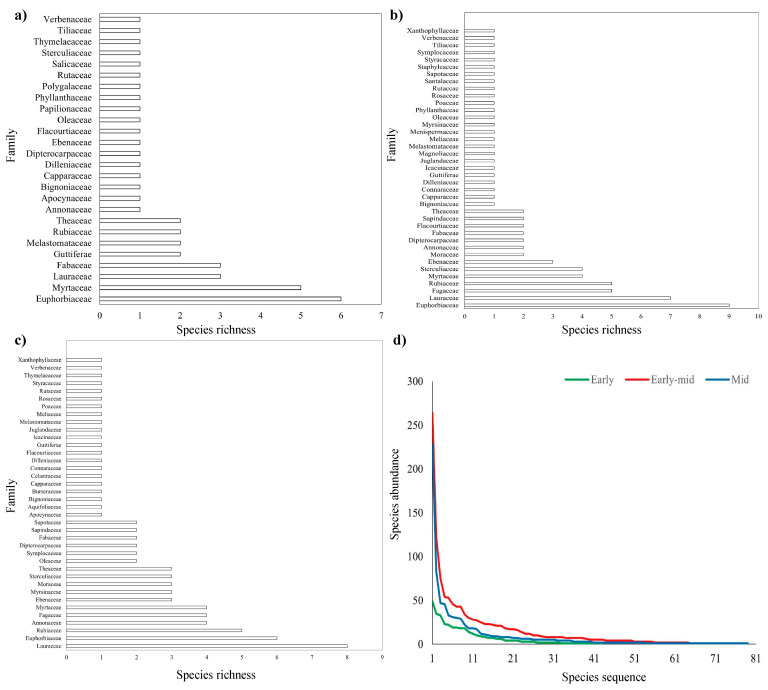
Plant community composition of tropical rainforests at early (**a**), early-mid (**b**), mid (**c**) successional stage, and species-abundance curves (**d**). Details of plant family names and their community composition can be seen in Table S2.

In the early successional stage, the tropical lowland rainforest contained 26 families, 40 genera, and 43 species of trees (Table S1a). There were 8 families with more than one species among them, accounting for 30.77% of all families (Table S2a, Figure 1a). The top three families with the most abundant genus-species ratios were Euphorbiaceae, Myrtaceae, and Lauraceae, whose genus-species ratios were 5:6, 4:5, and 2:3, respectively. In the early-mid successional stage, the tropical rainforest had 39 families, 63 genera, and 76 species of trees (Table S1b). There were 14 families with several species among them, accounting for 35.90% of the total number of families (Table S2b, Figure 1b). Among them, the top three families with the most abundant genus-species ratio were Ebenaceae, Myrtaceae, and Moraceae, and it included 38 families, 67 genera, and 79 species of trees at the mid-stage successional stage (Table S1c). There were 17 families with more than one species among them, accounting for 44.74% of all families (Table S2c, Figure 1c). The top three families with the most abundant genus-species ratio were Ebenaceae, Myrsinaceae, and Oleaceae. It can be seen that the complexity of tropical lowland rainforest plant communities has gradually increased as rainforest succession has progressed.

The species abundance was sorted from largest to smallest as the abscissa, and the species abundance as the ordinate to make a species-abundance curve (Figure 1d). The curve of tropical rainforests in early-mid successional stage was higher than that in the mid stage and early stage. This demonstrated that the tropical rainforest plant community was thriving in the early-mid stage of succession. The abundance of several species had decreased throughout the mid stage of succession, but the community’s development had gradually stabilized.

**Figure 2:**
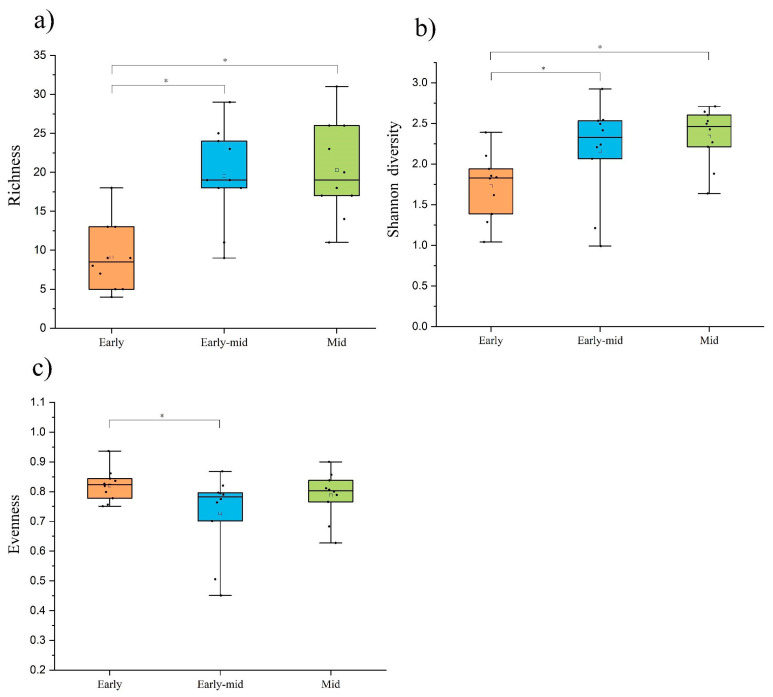
Plant community richness (**a**), Shannon diversity indexes (**b**), and evenness (**c**) differences at successional stages. Values are means ± standard error (*n* = 10). * *p* < 0.05 by one-tailed *t*-test.

With the succession of tropical rainforests, the plant richness and diversity index showed a rising trend, and the evenness index showed a trend of rising after the decline (Figure 2). The plant species richness and Shannon diversity indexes of early successional rainforests were significantly lower than those of early-mid and mid successional rainforests. As far as the Pielou evenness index is concerned, the early stage’s value was significantly greater than the early-mid stage, and there was no notable difference between early-mid and the mid stage.

### 2.2. Soil Properties among Different Successional Stages

**Figure 3:**
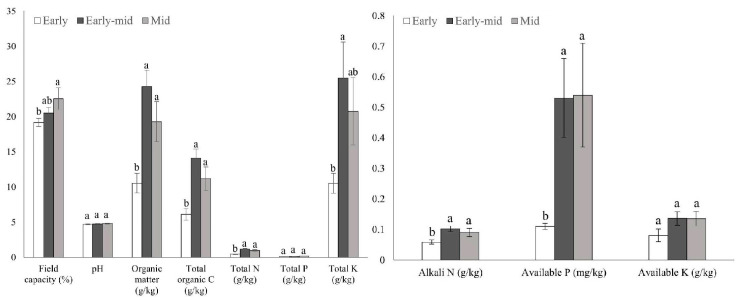
Soil properties differences at successional stages. Values are means ± standard error (*n* = 10). Different letters indicate *p* < 0.05 by Duncan’s multiple range test.

The three successional stages of tropical lowland rainforest had diverse soil physicochemical qualities (Figure 3). Specifically, in terms of soil physical properties, the field capacity was significantly lower in early stage (19.16 ± 0.57%) than in mid stage (22.54 ± 1.54%). The content of organic matter (OM) (10.54 ± 1.38 g/kg), total organic C (TOC) (6.11 ± 0.8 g/kg), total N (TN) (0.45 ± 0.05 g/kg), alkali N (AN) (58.9 ± 7.03 mg/kg), and available P (AP) (0.11 ± 0.01 mg/kg) were significantly lower in the early stage than in the early-mid (24.27 ± 2.30 g/kg, 14.08 ± 1.33 g/kg, 1.19 ± 0.09 g/kg, 102.27 ± 9.16 mg/kg, 0.53 ± 0.13 mg/kg) and mid stages (19.29 ± 2.84 g/kg, 11.19 ± 1.65 g/kg, 0.98 ± 0.12 g/kg, 90.88 ± 13.62 mg/kg, 0.54 ± 0.17 mg/kg). Between the early-mid and mid stages of succession, the soil nutrient level of the rainforest did not alter significantly, but in terms of quantity, except for total P (TP) and AP, all the other nutrients in the early-mid stage were greater than those in the mid stage.

### 2.3. Soil Bacterial Diversity and Networks

**Figure 4:**
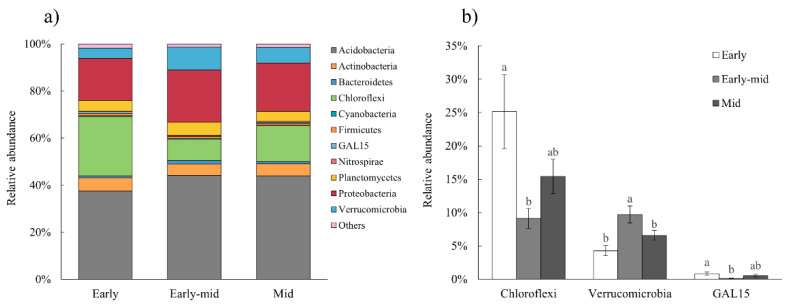
Relative abundance of the soil bacterial communities at the phylum level (**a**), and phyla with significant differences (**b**) at different successional stages. Only phyla with relative abundance >1% are shown. Values are means ± standard error (*n* = 10). Different letters indicate significant differences (*p* < 0.05) among the successional stages by Duncan’s multiple range test.

The relative abundances of soil bacterial communities in tropical lowland rainforests at various successional stages, as well as significant variations at the phylum level, were computed and examined (Figure 4). The relative abundances of soil microorganisms in different successional stages were discovered to be variable. Early-stage soil bacterial community composition differed obviously from early-mid and mid stage bacterial community makeup (Figure 4a). In the three successional stages of tropical lowland rainforest, the soil bacterial abundances of Chloroflexi, Verrucomicrobia, and GAL15 differed the most. The abundance of Chloroflexi changed as early > mid > early-mid, and there was a significant difference between the abundances in the early and early-mid stages (*p* < 0.05). The variation trend of Verrucomicrobia was early-mid > mid > early stage; during the succession of tropical lowland rainforest, the abundance of early-mid stage was significantly higher than that of early- and mid-stage (*p* < 0.05). With the development of rainforest succession, the changing trend of GAL15 is early > mid > early-mid stage, and its abundance change was significantly different (*p* < 0.05) between early and early-mid stages of succession (Figure 4b). Verrucomicrobia is a phylum of bacteria that was recently delineated, and there are few existing data on GAL15; only some taxonomic information can be found. Chloroflexi is a type of Galanz-negative bacteria that can produce energy through photosynthesis, and its response to sunlight is relatively active. This characteristic was consistent with the phenomenon that such bacteria were abundant in the early successional stage tropical lowland rainforest, with low canopy density and relatively more sunlight.

**Figure 5:**
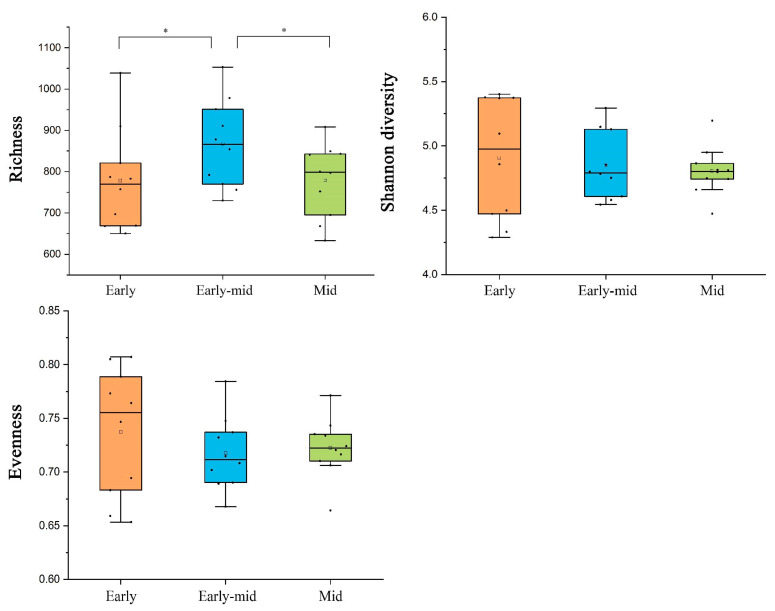
Soil bacterial diversity differences at successional stages. Values are means ± standard error (*n* = 10). * *p* < 0.05 by one-tailed *t*-test.

The soil bacterial richness increased significantly with time from the early successional stage to the early-mid stage, then decreased significantly with time to the mid successional stage (*p* < 0.05). The Shannon diversity decreased in the period from early successional stage to early-mid stage and then increased as time went by. Similarly, the evenness index first decreased drastically and then increased from early successional stage to mid stage (Figure 5).

**Figure 6:**
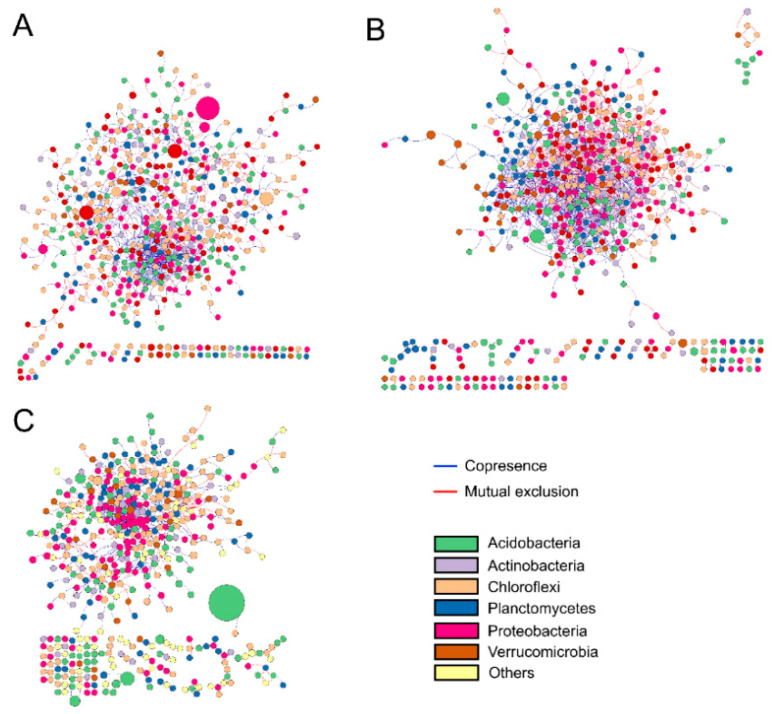
Co-occurrence network analysis of bacterial communities at early (**A**), early-mid (**B**), and mid (**C**) successional stage. Node sizes correspond to their relative abundance.

Co-occurrence network analysis was used to estimate bacterial interactions in the soil. A significantly higher network complexity was observed in the early-mid successional stage of tropical rainforest, with the average correlation per node increasing from 1.71 in the early successional stage to 2.19 in the early-mid stage, then decreasing to 1.53 in the mid stage (Table 2). Correspondingly, the total correlation number, including both positive and negative correlations, increased from 911 in the early successional stage to 1180 in the early-mid stage and decreased to 732 in the mid stage. The proportion of negative correlations increased from 27.9% in the early successional stage to 39.6% in the early-mid stage and subsequently reduced to 34.4% in the mid stage among the correlation associations calculated. The early successional stage had the largest number of modules with 66, followed by the mid stage with 63, and finally the early stage with 47. Overall, bacterial communities in early-mid successional stage tropical rainforest soils were more complex and had shorter average path length (Figure 6).

**Figure 7:**
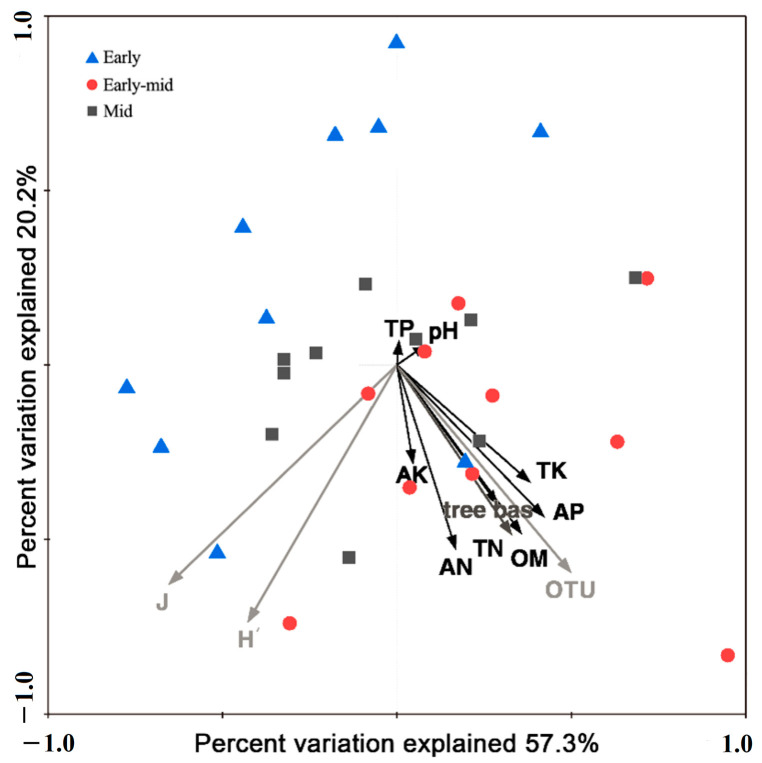
RDA ranking of the influence of tropical rainforest environmental factors on soil bacterial community changes in different successional stages. OM, organic matter; TN, total nitrogen; TP, total phosphorus; TK, total potassium; AN, available nitrogen; AP, available phosphorous; AK, available potassium; H′, Shannon diversity; J, Pielou evenness; tree bas, tree basal area; out, operational taxonomic units.

RDA analysis was used to determine the impact of environmental conditions on the soil bacterial community at each succession stage. The result explained 77.5% of the impact, with RDA axis 1 accounting for 57.3% of the total (Figure 7). Collectively, the impact of environmental factors on the soil bacterial community in the early stage of succession was milder than that in the early-mid and mid stages of succession. The effect of soil nutrient content on the number of OTU was greater than that of J and H′. The factors OM, TN, AN, and AP had a stronger positive impact on soil bacterial communities.

### 2.4. Niche Overlap and Interspecific Linkages of Leguminous Trees

Leguminous trees have specific effects on soil N concentration due to their distinct biological properties. The species and quantity of legume species in tropical lowland rainforest were tallied, and their niche overlap and interspecific connections in plant communities were investigated. In the early successional stage, 3 genera and 3 species of leguminous trees were found in the plant community, namely *Albizia attopeuensis*, *Ormosia semicastrata*, and *Peltophorum pterocarpum*. Leguminous trees and other plants in tropical lowland rain forests formed a total of 129 plant pairs, of which 36 pairs had niche overlap, accounting for 27.91% of the total, and 1 pair had an overlap index of 1, accounting for 0.78% of the total, which was Ppte-Vpie (Figure S1a). In the early-mid successional stage, there were 2 genera and 2 species of leguminous trees, which were *A. attopeuensis* and *P. pterocarpum.* There were 152 pairs of plant species composed of Fabaceae and other plants, among which 56 pairs had niche overlap, accounting for 36.84% of the total, and 8 pairs had an overlap index of 1, accounting for 5.26% of the total; they were Ppte-Atsa, Ppte-Dstr, Ppte-Egla, Ppte-Mcha, Ppte-Ptav, Ppte-Ptet, Ppte-Tcau and Ppte-Twal (Figure S1b). In the mid successional of tropical lowland rainforest, there were 2 genera and 2 species of Fabaceae, namely *A. attopeuensis* and *Sindora glabra*. A total of 158 plant pairs had overlapping niches in 32 pairs, accounting for 20.25%, and 7 pairs had an overlap index of 1, accounting for 4.43%; they were Sgla-Ohai, Sgla-Fmic, Aatt-Cbre, Aatt-Step, Aatt-Twal, Aatt-Dstr, and Aatt-Pann (Figure S1c).

The changing trend of niche overlap ratio and overlap index of 1 of leguminous trees was the same across successional stages, increasing from the early to the early-mid stage and decreasing from the early-mid stage to the mid stage. The results of niche overlap can only be used to illustrate the use of growth resources, with no indication of the positive or negative interaction between plant species. Therefore, the interspecific connections of legumes found in the tropical lowland rainforest were investigated in this study.

The results of interspecific relationship analysis showed that in the early successional stage plant community, legumes had a significant positive correlation with 13 species of plants, accounting for 10.08% of the total, of which 9 pairs showed a positive correlation with *p* < 0.05, respectively, Aatt-Odio, Osem-Chor, Osem-Mdie, Osem-Ppte, Osem-Prub, Osem-Vpie, Osem-Wnut, Ppte-Ccoc, and Ppte-Osem; 2 pairs showed a positive correlation of *p* < 0.01, respectively, Aatt-Agha and Aatt-Gobl; 2 pairs showed a positive correlation of *p* < 0.001, Ppte-Spro and Ppte-Vpie (Figure S2a). In the tropical lowland rainforest plant community of the early-mid stage of natural succession, there was a significant positive correlation between leguminous trees and 20 species of plants, accounting for 13.16% of the total: Ppte-Anchi and Aatt-Aoli showed a positive correlation of *p* < 0.05; Aatt-Schu and Aatt-Cfur showed a positive correlation of *p* < 0.01; Aatt-Agha, Aatt-Byun, Aatt-Cafor, Aatt-Chai, Aatt-Hpha, Aatt-Slau, Aatt-Dhow, Ppte-Clae, Ppte-Atsa, Ppte-Dstr, Ppte-Egla, Ppte-Mcha, Ppte-Ptav, Ppte-Ptet, Ppte-Tcau, and Ppte-Twal showed a positive correlation of *p* < 0.001 (Figure S2b). In the mid successional tropical lowland rainforest, leguminous trees had a significant positive correlation with 18 plants, accounting for 11.39% of the total, of which 2 pairs showed a positive correlation with *p* < 0.05, namely Sgla-Dhow and Aatt-Fruk; 5 pairs showed a positive correlation of *p* < 0.01, Aatt-Mpom, Aatt-Mlig, Aatt-Adio, Sgla-Sglo, and Sgla-Xhai; 11 pairs showed a positive correlation of *p* < 0.001, Aatt-Cbre, Aatt-Dstr, Aatt-Pann, Aatt-Step, Aatt-Twal, Aatt-Anchi, Aatt-Rdum, Sgla-Fmic, Sgla-Ohai, Sgla-Almon, and Sgla-Kbai (Figure S2c).

Overall, the positive relationship between legumes and other plants was in the order of early-mid stage > mid > early stage, and this trend was consistent with the changes in niche overlap.

## 3. Discussion

### 3.1. The Development Direction of Plant Community Diversity and Interspecific Relationship of Leguminous Trees

Forests with different disturbance histories have different vegetation recovery speeds and patterns. Natural disturbances such as floods or volcanic eruptions can affect forest ecosystems for hundreds of years, and these disturbances may cause long-term changes in biodiversity patterns by causing the extinction of some plant species [26,27]. For forests disturbed by slash-and-burn cultivation, the plant types that appeared in the early stage of forest succession were basically herbs, and then a forest plant community with arbor plants as the dominant species gradually developed. As time went by, after the early stage of succession, the pioneer tree species and sun plants among the arbor species gradually appeared and developed, and the forest plant community gradually recovered to the state before the destruction [28]. In our study, forests were mainly disturbed by human logging of timber. In our study, the results showed that the species richness and diversity of arbor plants in tropical lowland rainforest continued to increase with the process of rainforest succession, and the results of early-mid and mid successional stages were significantly higher (*p* < 0.05) than those in the early successional stage (Table 2). Re-establishment of tropical forest to approximate pre-disturbance levels typically requires 20–200 years [29]; during this process the forest plant community develops towards the climax community. Along the entire succession process, the plant community will first go through a period of fast development in species richness and diversity [30], during which the environment will change in a way that benefits the present dominating species’ survival. After that, the forest plant community will go through a stage of competition. In this stage, various species in the community will compete for living space, soil moisture, soil nutrients, etc., so that the dominance of the species that adapt to the competition increases, and the species that do not adapt to the competition are pushed out of the community [31]. Finally, the forest plant community will enter a relatively stable stage. The composition and structure of species in the community tend to be in a balance status through competition, and the community and the environment have relative stability. In our study, the forest with the longest succession development time is only in the mid stage of succession, and the plant community in the forest has not yet reached the state of the climax community; in other words, it has not entered the above-mentioned relatively stable stage. Therefore, the species richness and diversity of the plant communities in our study showed a continuous increase with the development of rainforest succession. As far as the Pielou evenness index is concerned, the early stage’s value was significantly greater than the early-mid stage, and there was no notable difference between early-mid and the mid stage. This may be because the composition and structure of rainforest plants are relatively simple in the early stages of succession, the utilization of environmental resources by plant communities is insufficient, and the development status of each species is relatively similar, resulting in a high evenness index. Afterwards, with the continuous development of plant communities, each species’ use of environmental resources differed, resulting in varied development status, and the dominant species progressively emerged, leading in a fall in the evenness index.

Legumes (Fabaceae species) are thought to have some effect on soil N content. Our results showed that there were 4 genera (*Albizia*, *Ormosia*, *Peltophorum*, *Sindora*) and 4 species (*A. attopeuensis*, *O. semicastrata*, *P. pterocarpum*, *S. glabra*) of Fabaceae species in the tropical lowland rainforest; all of them were regarded as N-fixing legumes because plants in *Albizia* and *Ormosia* can have a mutualism relationship with rhizobia to have nitrogen fixation ability [32], and plants in *Peltophorum* and *Sindora* were also determined to be N-fixing trees [33,34]. In this experiment, analysis of the relationship between species of leguminous trees showed that the positive relationship between legume tree species and other plants was in the order of early-mid stage > mid > early stage, and this trend was consistent with the changes in niche overlap (Figure S1a–Figure S2c). This indicated that the legume tree species had a better growth condition in the early-mid of the tropical lowland rainforest succession stage and had a positive correlation between species, and could be considered to achieve better nitrogen fixation.

### 3.2. Soil Properties and Soil Bacteria Community Characteristics along the Succession

The results showed that in terms of rainforest soil physical properties, the field capacity was significantly lower in the early stage than in the mid stage. For soil nutrient content, except for P, all the results of nutrient content observations were early-mid stage > mid stage > early stage (Figure 3). Along forest succession, soil nutrient content will change due to changes of plant communities. Higher plant diversity leads to more nutrient input into forest soils; at the same time, the decomposition of plant litter can also increase soil nutrient content [10]. The overall increase in soil nutrient content will prompt plants to absorb nutrients. Under the same environmental factors (rock decomposition and rain leaching can increase soil nutrient content), due to the existence of nitrogen-fixing plants, soil N elements are more than P elements; therefore, the loss of P in rainforest soil is larger than that of N. This could be the reason why the content of P in the early-mid successional stage is lower than that in the mid stage.

Soil bacterial richness changed significantly along with forest succession progress, but species diversity and evenness did not (Figure 5). RDA analysis showed that the factors OM, TN, AN, and AP had a stronger positive impact on soil bacterial communities OTU amount, and this was consistent with the results of soil nutrient content, which was that the early-mid successional stage tropical rainforest with the highest soil nutrient content had significantly higher soil bacterial richness than the early and mid stage. In our experiments, soil bacterial community richness increased with soil nutrient content, which is similar to previous studies [35].

### 3.3. Effects of Natural Succession on Bacterial Co-Occurrence Network Interactions

Despite the relationships between microorganisms being extremely complex and that soil bacteria interactions are difficult to accurately measure and quantify, co-occurrence network analysis provided a good method to explore the interactive effects of bacteria [36,37]. We proposed a prediction that the bacterial interactions are strengthened in the early-mid successional stage, generating more complex networks. There are several pieces of evidence that confirm our prediction. First, bacterial interactions became more connected and clustered, especially in the early-mid successional stage network (Figure 6, Table 2). Second, more negative connections were observed in the early-mid successional stage than the early and mid stage network (Table 2). Third, the lowest number of modules was observed in the early successional stage network, but the number increased substantially in early-mid and mid restoration stage networks (Table 2).

The negative correlation could be used to infer relationships between bacterial species, especially competitive relationships [36]. In our research, the negative correlation first increased and then decreased along the successional stages; this might indicate that the competitive relationships between bacterial populations increase in the early to early-mid successional stage, and then gradually decrease in the early-mid to the mid restoration stage. Lower negative correlations and more isolated species in the network under early- and mid-successional stages may indicate that these particular taxa fill specific fragmented niche spaces, for which no direct competitors exist [38]. Higher network complexity under the early-mid successional stage might be explained by the increase of nutrients in the soil (Figure 3), providing more opportunities for different species to interact with each other [39].

## 4. Materials and Methods

### 4.1. Study Site and Experimental Design

The study site is located in the Ganshiling Nature Reserve (2103.44 ha) of Hainan Island (109°34′ E–109°42′ E, 18°20′ N–18°21′ N), which is the southernmost provincial nature reserve in China. Tropical lowland rainforest is the type of vegetation found here. Hainan Island is located on the tropics’ northern edge, is an area of tropical marine monsoon climate, with an average annual rainfall of 1800 mm and a mean annual temperature of 24.5 °C [18]. The field soil is Oxisol in the USDA Soil Taxonomy.

**Figure 8:**
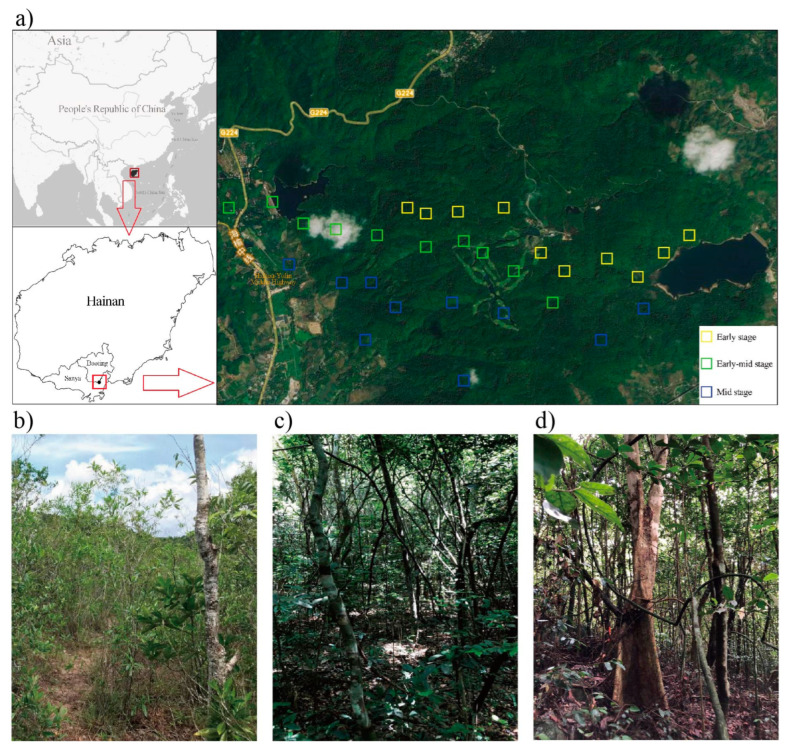
Location of the Hainan Island and the study site (**a**); plant growth status of tropical lowland rainforest in the early (**b**), early-mid (**c**) and mid (**d**) successional stages.

Three tropical lowland rainforests disturbed by deforestation with different natural secondary succession stages were selected in Hainan, China, namely early successional stage (33 years), early-mid successional stage (60 years), and mid successional stage (73 years) according to the definition of previous scholars [25,26,27,28,29,30,31,32,33,34,35,36,37,38,39,40]. Within each of the 3 kinds of successional stage rainforest, 10 plots (20 × 20 m) were established for soil sampling and plant community surveying. All of the 30 plots (3 successional stages × 10 plots) were on a similar slope position. To guarantee spatial heterogeneity, each of the plots (30 in total, 20 × 20 m) were separated from each other by at least 500 m (Figure 8).

### 4.2. Plant Community Surveying and Soil Sampling

In the period June–August 2018, the sample plots’ tree species were identified, and each tree was examined. The tree height (Vertex Laser Ultrasonic Tree Height Range Finder, Haglof, Dalarna, Sweden) and diameter at breast height (≥3 cm) (tape measure) were measured and recorded.

In each site, 3 soil samples, each of 0–20 cm, 20–40 cm, 40–60 cm, 60–80 cm, and 80–100 cm depth were collected with 100 cm^3^ cutting-ring method soil sample containers after removing the plant litter layer in an S-shaped pattern. A total of 450 cutting-ring method soil sample containers with collected soil (3 successional stages × 10 plots × 5 depth × 3 samples) were used to measure soil physical properties. At the same time, 3 soil samples in each plot of each depth were collected and mixed to form a final composite sample, kept on ice. All the mixed soil samples were passed through a 2 mm sieve to remove litter, roots and rocks. Then, each of the total 30 mixed soil samples was divided into two parts: one of them was stored at −80 °C for DNA extraction, the other one was stored at 4 °C for soil property measurements. All soil sample collections were completed within the same week.

### 4.3. Analysis of Soil Physical and Chemical Properties

Soil moisture content was measured by drying weighing (105 °C), and field capacity was measured by the cutting-ring method. Soil pH was measured by a pH meter using the soil-water slurry (1:5) method. An elemental analyzer (Costech ECS 4024 CHNSO, Picarro, CA, USA) was used to test total organic carbon (TOC) and total nitrogen (TN) of soil. The automatic chemical analyzer (Smartchem 300, AMS, Bergamo, Italy) was used to measured soil total phosphorus (TP) by Mo-Sb colorimetric method. The Kjeldahl method analyzer (Kjeldahl 2300, Foss, Denmark) was used to examine alkali nitrogen (AN). Soil available phosphorus (AP) was determined with the ultraviolet spectrophotometry method. Soil total potassium (TK) was measured by a flame photometer (M410, Sherwood, UK). Atomic absorption spectrophotometry was employed to measure soil available potassium (AK).

### 4.4. Soil Samples DNA Extraction, PCR Amplification, and Sequencing

Genomic DNA was extracted using a MOBIO Power Soil^®^ DNA Isolation Kit (MOBIO Laboratories, Carlsbad, CA, USA) according to the manufacturer’s instructions. The concentration and purity were measured using the NanoDrop One (Thermo Fisher Scientific, MA, USA). 16S rRNA genes of distinct regions V4-V5 were amplified used specific primer 515F and 907R synthesized by Invitrogen (Invitrogen, Carlsbad, CA, USA) with 12bp barcode. PCR amplification, gel extraction, and sequence processing were operated as described previously by Hu et al. [18].

### 4.5. Data Analysis

Statistical analyses of the data were carried out using SPSS 11.5 for Windows (SPSS Inc., Chicago, IL, USA). RDA ordination was calculated by Origin 2021 for Windows (OriginLab, Northampton, MA, USA).

In this section, the plant data of 10 plots in each successional stage was combined to calculate the final results of different stages. Niche overlap was used to explore the similarity of resource utilization capabilities of two plant species, and interspecific association was used to describe the attributes of repulsion (negative correlation) or attraction (positive correlation) between two species. The analysis of plant niche overlap and interspecific associations were performed using packages spaa, permute, lattice, vegan, psych, and ggplot2 (R Core Team, 2019) written with the R language (https://cran.r-project.org/web/packages/spaa/index.html; https://cran.r-project.org/web/packages/permute/index.html; https://cran.r-project.org/web/packages/lattice/index.html; https://cran.r-project.org/web/packages/vegan/index.html; https://cran.r-project.org/web/packages/psych/index.html; https://cran.r-project.org/web/packages/ggplot2/index.html) accessed 1 December 2021 on the basis of the importance value of each plant species.

Co-occurrence network analysis was used to infer the microbial interactions. All the principles, formulas, and the meanings represented by the formulas are detailed in Deng [41], and a pipeline is available at http://ieg2.ou.edu/MENA, accessed 1 December 2021. The network topologies were calculated in R environment (http://www.r-project.org, accessed 1 December 2021) using the “igraph” package.

## 5. Conclusions

In summary, during the secondary succession of tropical lowland rainforests, plant richness and species diversity increased significantly. Nitrogen-fixing leguminous trees developed best in the early-mid stages of succession and had significant positive correlations with many other species within the successional plant communities. Soil nutrient content and soil bacterial richness were highest in the early-mid stages of succession, while OM, TN, AN, and AP had a stronger positive impact on soil bacterial communities. With the advancement of forest succession, the negative correlation between soil bacterial species decreased, and the community stability increased, which was reflected in network analysis. Thus, this research can act as a framework that provides insight into the synergistic relationship between plant community–soil physicochemical properties–soil bacterial community during natural secondary tropical lowland rainforest succession. This may help us to better understand the plant growth status during succession and how interspecific relationships of soil bacterial communities can be affected by altering soil nutrient content. Future studies are required to illustrate how did the beta diversity of plants and soil bacteria change during forest succession, and whether they also followed the Plant-Soil-Soil-bacteria influence pattern.

## Figures and Tables

**Table 1:** General situation of stand structures at different successional stages.

Succession Stage	Early	Early-Mid	Mid
Basal area (m^2^/ha)	4.74 ± 1.05 ^c^	36.21 ± 2.52 ^b^	42.99 ± 2.22 ^a^
Stem density (tree/ha)	917.50 ± 143.47 ^c^	3165.00 ± 247.91 ^a^	2057.50 ± 200.52 ^b^
Mean height (m)	3.75 ± 0.22 ^b^	10.52 ± 0.30 ^a^	10.60 ± 0.43 ^a^
Maximum height (m)	6.46 ± 0.50 ^b^	22.07 ± 1.11 ^a^	23.25 ± 1.58 ^a^

Values are means ± standard error (*n* = 10). Significance is based on the Duncan’s multiple range test. Means followed by a common letter are not significantly different at the 5% level of significance.

**Table 2:** Network properties of the bacterial co-occurrence networks at early, early-mid, and mid successional stages.

	Early	Early-Mid	Mid
Total degree	532	539	477
Total correlation	911	1180	732
Positive correlation	657 (72.1%)	713 (60.4%)	480 (65.6%)
Negative correlation	254 (27.9%)	467 (39.6%)	252 (34.4%)
Average correlation per node	1.71	2.19	1.53
Average path length	7.57	4.7	6.33
Average clustering coefficient	0.3	0.21	0.29
Modularity	0.751	0.626	0.789
Number of modules	47	66	63

## Data Availability

Not applicable.

## Supplementary Materials

The following supporting information can be downloaded at: https://www.mdpi.com/article/10.3390/plants11101344/s1, Figure S1a: Niche overlap of leguminous trees in early successional stages; Figure S1b: Niche overlap of leguminous trees in early-mid successional stages; Figure S1c: Niche overlap of leguminous trees in mid successional stages; Figure S2a: Pearson’s significance and correlation coefficients of leguminous trees in early successional stages; Figure S2b: Pearson’s significance and correlation coefficients of leguminous trees in early-mid successional stages; Figure S2c: Pearson’s significance and correlation coefficients of leguminous trees in mid successional stages; Table S1a: The plant Family, Genus, Species and name abbreviations of early successional stage; Table S1b: The plant Family, Genus, Species and name abbreviations of early-mid successional stage; Table S1c: The plant Family, Genus, Species and name abbreviations of mid successional stage; Table S2a: Plant composition of families, genera and species in early succession stage; Table S2b: Plant composition of families, genera and species in early-mid succession stage; Table S2c: Plant composition of families, genera and species in mid succession stage.
