# Supplementary material for: Secondary Succession Altered the Diversity and Co-Occurrence Networks of the Soil Bacterial Communities in Tropical Lowland Rainforests
Source: Plants (Basel). 2022 May 19;11(10):1344. doi: 10.3390/plants11101344 (PMC9148129; doi:10.3390/plants11101344)
Supplement: Supplementary file 1 [file plants-11-01344-s001.zip › plants-1651094-supplementary.pdf]

Table S1. a The plant Family, Genus, Species and name abbreviations of early successional stage.

	Abbreviation	Species name	Genus	Family
1	Aatt	*Albizia attopeuensis*	*Albizia*	Fabaceae
2	Adio	*Aporusa dioica*	*Aporusa*	Euphorbiaceae
3	Agha	*Antidesma ghaesembilla*	*Antidesma*	Euphorbiaceae
4	Amon	*Antidesma montanum*	*Antidesma*	Euphorbiaceae
5	Avil	*Aporusa villosa*	*Aporusa*	Phyllanthaceae
6	Ccoc	*Cratoaylum cochinchinense*	*Cratoxylum*	Guttiferae
7	Cfor	*Crateva formosensis*	*Crateva*	Capparaceae
8	Chor	*Canthium horridum*	*Canthium*	Rubiaceae
9	Cjap	*Camellia japonica*	*Camellia*	Theaceae
10	Cpor	*Cinnamomum porrectum*	*Cinnamomum*	Lauraceae
11	Dchu	*Diospyros chunii*	*Diospyros*	Ebenaceae
12	Dgra	*Decaspermum gracilentum*	*Decaspermum*	Myrtaceae
13	Dtur	*Dillenia turbinata*	*Dillenia*	Dilleniaceae
14	Fruk	*Flacourtia rukam*	*Flacourtia*	Flacourtiaceae
15	Gobl	*Garcinia oblongifolia*	*Garcinia*	Guttiferae
16	Gwri	*Glochidion wrightii*	*Glochidion*	Euphorbiaceae
17	Hang	*Heritiera angustata*	*Heritiera*	Sterculiaceae
18	Kbai	*Koilodepas bainanense*	*Koilodepas*	Euphorbiaceae
19	Mchi	*Machilus chinensis*	*Machilus*	Lauraceae
20	Mchu	*Microcos chungii*	*Microcos*	Tiliaceae
21	Mdie	*Millettia dielsiana*	*Millettia*	Papilionaceae
22	Mlig	*Memecylon ligustrifolium*	*Memecylon*	Melastomataceae
23	Mpom	*Machilus pomifera*	*Machilus*	Lauraceae
24	Msan	*Melastoma sanguineum*	*Melastoma*	Melastomataceae
25	Odio	*Olea dioica*	*Olea*	Oleaceae
26	Osem	*Ormosia semicastrata*	*Ormosia*	Fabaceae
27	Pemb	*Phyllanthus emblica*	*Phyllanthus*	Euphorbiaceae
28	Ppte	*Peltophorum pterocarpum*	*Peltophorum*	Fabaceae
29	Prub	*Psychotria rubra*	*Psychotria*	Rubiaceae
30	Rdum	*Rhodamnia dumetorum*	*Rhodamnia*	Myrtaceae
31	Rfro	*Radermachera frondosa*	*Radermachera*	Bignoniaceae
32	Rtom	*Rhodomyrtus tomentosa*	*Rhodomyrtus*	Myrtaceae
33	Shai	*Syzygium hainanense*	*Syzygium*	Myrtaceae
34	Smyr	*Salix myrtillacea*	*Salix*	Salicaceae
35	Spro	*Saccopetalum prolificum*	*Saccopetalum*	Annonaceae
36	Ssup	*Schima superba*	*Schima*	Theaceae
37	Step	*Syzygium tephrodes*	*Syzygium*	Myrtaceae
38	Vman	*Vatica mangachapoi*	*Vatica*	Dipterocarpaceae
39	Vpie	*Vitex pierreana*	*Vitex*	Verbenaceae
40	Wnut	*Wikstroemia nutans*	*Wikstroemia*	Thymelaeaceae
41	Wpub	*Wrightia pubescens*	*Wrightia*	Apocynaceae

42	Xhai	*Xanthophyllum hainanense*	*Xanthophyllum*	Polygalaceae
43	Zavi	*Zanthoxylum avicennae*	*Zanthoxylum*	Rutaceae

Table S1. b The plant Family, Genus, Species and name abbreviations of early-mid successional stage.

	Abbreviation	Species name	Genus	Family
1	Aatt	*Albizia attopeuensis*	*Albizia*	Fabaceae
2	Aden	*Ardisia densilepidotula*	*Ardisia*	Myrsinaceae
3	Agha	*Antidesma ghaesembilla*	*Antidesma*	Euphorbiaceae
4	Ahai	*Antidesma hainanense*	*Antidesma*	Euphorbiaceae
5	Alau	*Albertisia laurifolia*	*Albertisia*	Menispermaceae
6	Almon	*Alphonsea monogyna*	*Alphonsea*	Annonaceae
7	Amchi	*Amesiodendron chinense*	*Amesiodendron*	Sapindaceae
8	Anchi	*Antirhea chinensis*	*Antirhea*	Rubiaceae
9	Anmon	*Antidesma montanum*	*Antidesma*	Euphorbiaceae
10	Aoli	*Acronychia oligophlebia*	*Acronychia*	Rutaceae
11	Atsa	*Amoora tsangii*	*Amoora*	Meliaceae
12	Avil	*Aporusa villosa*	*Aporusa*	Phyllanthaceae
13	Bins	*Bridelia insulana*	*Bridelia*	Euphorbiaceae
14	Blon	*Beilschmiedia longipetiolata*	*Beilschmiedia*	Lauraceae
15	Byun	*Beilschmiedia yunnanensis*	*Beilschmiedia*	Lauraceae
16	Cafor	*Castanopsis formosana*	*Castanopsis*	Fagaceae
17	Cbla	*Cyclobalanopsis blakei*	*Cyclobalanopsis*	Fagaceae
18	Cfur	*Camellia furfuracea*	*Camellia*	Theaceae
19	Chai	*Castanopsis hainanensis*	*Castanopsis*	Fagaceae
20	Cjuc	*Castanopsis jucunda*	*Castanopsis*	Fagaceae
21	Clae	*Croton laevigatus*	*Croton*	Euphorbiaceae
22	Cmet	*Cryptocarya metcalfiana*	*Cryptocarya*	Lauraceae
23	Cpor	*Cinnamomum porrectum*	*Cinnamomum*	Lauraceae
24	Crfor	*Crateva formosensis*	*Crateva*	Capparaceae
25	Dchu	*Diospyros chunii*	*Diospyros*	Ebenaceae
26	Dhow	*Diospyros howii*	*Diospyros*	Ebenaceae
27	Dstr	*Diospyros strigosa*	*Diospyros*	Ebenaceae
28	Dtur	*Dillenia turbinata*	*Dillenia*	Dilleniaceae
29	Efen	*Engelhardia fenzelii*	*Engelhardtia*	Juglandaceae
30	Egla	*Ellipanthus glabrifolius*	*Ellipanthus*	Connaraceae
31	Fcon	*Ficus concinna*	*Ficus*	Moraceae
32	Fdep	*Fagerlindia depauperata*	*Fagerlindia*	Rubiaceae
33	Fvar	*Ficus variolosa*	*Ficus*	Moraceae
34	Gchi	*Goniothalamus chinensis*	*Goniothalamus*	Annonaceae
35	Gcoc	*Glochidion coccineum*	*Glochidion*	Euphorbiaceae
36	Glob	*Gonocaryum lobbianum*	*Gonocaryum*	Icacinaceae
37	Gobl	*Garcinia oblongifolia*	*Garcinia*	Guttiferae
38	Gwri	*Glochidion wrightii*	*Glochidion*	Euphorbiaceae
39	Hang	*Heritiera angustata*	*Heritiera*	Sterculiaceae
40	Hexa	*Hopea exalata*	*Hopea*	Dipterocarpaceae
41	Hpar	*Heritiera parvifolia*	*Heritiera*	Sterculiaceae

42	Hpha	*Homalium phanerophlebium*	*Homalium*	Flacourtiaceae
43	Kbai	*Koilodepas bainanense*	*Koilodepas*	Euphorbiaceae
44	Lchi	*Litchi chinensis*	*Litchi*	Sapindaceae
45	Lpha	*Laurocerasus phaeosticta*	*Laurocerasus*	Rosaceae
46	Lska	*Lithocarpus skanianus*	*Lithocarpus*	Fagaceae
47	Mcha	*Magnolia championii*	*Magnolia*	Magnoliaceae
48	Mchi	*Machilus chinensis*	*Machilus*	Lauraceae
49	Mchu	*Microcos chungii*	*Microcos*	Tiliaceae
50	Mhoo	*Mallotus hookerianus*	*Mallotus*	Euphorbiaceae
51	Mlig	*Memecylon ligustrifolium*	*Memecylon*	Melastomataceae
52	Mpom	*Machilus pomifera*	*Machilus*	Lauraceae
53	Odio	*Olea dioica*	*Olea*	Oleaceae
54	Ppte	*Peltophorum pterocarpum*	*Peltophorum*	Fabaceae
55	Prub	*Psychotria rubra*	*Psychotria*	Rubiaceae
56	Ptav	*Phoebe tavoyana*	*Phoebe*	Lauraceae
57	Ptet	*Prismatomeris tetrantra*	*Prismatomeris*	Rubiaceae
58	Rbot	*Reevesia botingensis*	*Reevesia*	Sterculiaceae
59	Rdum	*Rhodamnia dumetorum*	*Rhodamnia*	Myrtaceae
60	Rfro	*Radermachera frondosa*	*Radermachera*	Bignoniaceae
61	Sbra	*Syzygium brachythyrsum*	*Syzygium*	Myrtaceae
62	Sbum	*Staphylea bumalda*	*Staphylea*	Staphyleaceae
63	Schu	*Syzygium chunianum*	*Syzygium*	Myrtaceae
64	Shai	*Styrax hainanensis*	*Styrax*	Styracaceae
65	Slan	*Sterculia lanceolata*	*Sterculia*	Sterculiaceae
66	Slau	*Sarcosperma laurinum*	*Sarcosperma*	Sapotaceae
67	Spoi	*Symplocos poilanei*	*Symplocos*	Symplocaceae
68	Ssae	*Scolopia saeva*	*Scolopia*	Flacourtiaceae
69	Ssup	*Schima superba*	*Schima*	Theaceae
70	Step	*Syzygium tephrodes*	*Syzygium*	Myrtaceae
71	Swal	*Scleropyrum wallichianum*	*Scleropyrum*	Santalaceae
72	Tcau	*Themeda caudata*	*Themeda*	Poaceae
73	Twal	*Tarennoidea wallichii*	*Tarennoidea*	Rubiaceae
74	Vman	*Vatica mangachapoi*	*Vatica*	Dipterocarpaceae
75	Vpie	*Vitex pierreana*	*Vitex*	Verbenaceae
76	Xhai	*Xanthophyllum hainanense*	*Xanthophyllum*	Xanthophyllaceae

Table S1. c The plant Family, Genus, Species and name abbreviations of mid successional stage.

	Abbreviation	Species name	Genus	Family
1	Aatt	*Albizia attopeuensis*	*Albizia*	Fabaceae
2	Aden	*Ardisia densilepidotula*	*Ardisia*	Myrsinaceae
3	Adio	*Aporusa dioica*	*Aporusa*	Euphorbiaceae
4	Almon	*Alphonsea monogyna*	*Alphonsea*	Annonaceae
5	Amchi	*Amesiodendron chinense*	*Amesiodendron*	Sapindaceae
6	Anchi	*Antirhea chinensis*	*Antirhea*	Rubiaceae
7	Aner	*Ardisia nervosa*	*Ardisia*	Myrsinaceae
8	Anmon	*Antidesma montanum*	*Antidesma*	Euphorbiaceae
9	Aoli	*Acronychia oligophlebia*	*Acronychia*	Rutaceae
10	Aqui	*Ardisia quinquegona*	*Ardisia*	Myrsinaceae
11	Asin	*Aquilaria sinensis*	*Aquilaria*	Thymelaeaceae
12	Atsa	*Amoora tsangii*	*Amoora*	Meliaceae
13	Blon	*Beilschmiedia longipetiolata*	*Beilschmiedia*	Lauraceae
14	Cafor	*Castanopsis formosana*	*Castanopsis*	Fagaceae
15	Calb	*Canarium album*	*Canarium*	Burseraceae
16	Cbla	*Cyclobalanopsis blakei*	*Cyclobalanopsis*	Fagaceae
17	Cbre	*Callicarpa brevipes*	*Callicarpa*	Verbenaceae
18	Cfur	*Camellia furfuracea*	*Camellia*	Theaceae
19	Chai	*Castanopsis hainanensis*	*Castanopsis*	Fagaceae
20	Clae	*Croton laevigatus*	*Croton*	Euphorbiaceae
21	Cmet	*Cryptocarya metcalfiana*	*Cryptocarya*	Lauraceae
22	Cole	*Camellia oleifera*	*Camellia*	Theaceae
23	Cpor	*Cinnamomum porrectum*	*Cinnamomum*	Lauraceae
24	Crfor	*Crateva formosensis*	*Crateva*	Capparaceae
25	Dchu	*Diospyros chunii*	*Diospyros*	Ebenaceae
26	Ddub	*Diplospora dubia*	*Diplospora*	Rubiaceae
27	Dhai	*Decaspermum hainanense*	*Decaspermum*	Myrtaceae
28	Dhow	*Diospyros howii*	*Diospyros*	Ebenaceae
29	Dstr	*Diospyros strigosa*	*Diospyros*	Ebenaceae
30	Dtur	*Dillenia turbinata*	*Dillenia*	Dilleniaceae
31	Efen	*Engelhardia fenzelii*	*Engelhardtia*	Juglandaceae
32	Egla	*Ellipanthus glabrifolius*	*Ellipanthus*	Connaraceae
33	Fmic	*Ficus microcarpa*	*Ficus*	Moraceae
34	Fruk	*Flacourtia rukam*	*Flacourtia*	Flacourtiaceae
35	Fvar	*Ficus variolosa*	*Ficus*	Moraceae
36	Ggar	*Goniothalamus gardneri*	*Goniothalamus*	Annonaceae
37	Glob	*Gonocaryum lobbianum*	*Gonocaryum*	Icacinaceae
38	Gobl	*Garcinia oblongifolia*	*Garcinia*	Guttiferae
39	Hang	*Heritiera angustata*	*Heritiera*	Sterculiaceae
40	Hexa	*Hopea exalata*	*Hopea*	Dipterocarpaceae
41	Hpar	*Heritiera parvifolia*	*Heritiera*	Sterculiaceae
42	Itri	*Ilex triflora*	*Ilex*	Aquifoliaceae

43	Kbai	*Koilodepas bainanense*	*Koilodepas*	Euphorbiaceae
44	Lchi	*Litchi chinensis*	*Litchi*	Sapindaceae
45	Lcom	*Lindera communis*	*Lindera*	Lauraceae
46	Lelo	*Litsea elongata*	*Litsea*	Lauraceae
47	Lpha	*Laurocerasus phaeosticta*	*Laurocerasus*	Rosaceae
48	Lska	*Lithocarpus skanianus*	*Lithocarpus*	Fagaceae
49	Mchi	*Machilus chinensis*	*Machilus*	Lauraceae
50	Mhoo	*Mallotus hookerianus*	*Mallotus*	Euphorbiaceae
51	Mlig	*Memecylon ligustrifolium*	*Memecylon*	Melastomataceae
52	Mpom	*Machilus pomifera*	*Machilus*	Lauraceae
53	Msua	*Melodinus suaveolens*	*Melodinus*	Apocynaceae
54	Odio	*Olea dioica*	*Olea*	Oleaceae
55	Ohai	*Olea hainanensis*	*Olea*	Oleaceae
56	Pann	*Pouteria annamensis*	*Pouteria*	Sapotaceae
57	Ppis	*Popowia pisocarpa*	*Popowia*	Annonaceae
58	Prub	*Psychotria rubra*	*Psychotria*	Rubiaceae
59	Ptav	*Phoebe tavoyana*	*Phoebe*	Lauraceae
60	Ptet	*Prismatomeris tetrantra*	*Prismatomeris*	Rubiaceae
61	Rdum	*Rhodamnia dumetorum*	*Rhodamnia*	Myrtaceae
62	Rfro	*Radermachera frondosa*	*Radermachera*	Bignoniaceae
63	Rthy	*Reevesia thyrsoidess*	*Reevesia*	Sterculiaceae
64	Schu	*Syzygium chunianum*	*Syzygium*	Myrtaceae
65	Sgla	*Sindora glabra*	*Sindora*	Fabaceae
66	Sglo	*Suregada glomerulata*	*Suregada*	Euphorbiaceae
67	Shai	*Styrax hainanensis*	*Styrax*	Styracaceae
68	Sili	*Streblus ilicifolius*	*Taxotrophis*	Moraceae
69	Slau	*Sarcosperma laurinum*	*Sarcosperma*	Sapotaceae
70	Smac	*Salacia macrophylla*	*Salacia*	Celastraceae
71	Spoi	*Symplocos poilanei*	*Symplocos*	Symplocaceae
72	Spro	*Saccopetalum prolificum*	*Saccopetalum*	Annonaceae
73	Spse	*Symplocos pseudobarberina*	*Symplocos*	Symplocaceae
74	Ssup	*Schima superba*	*Schima*	Theaceae
75	Step	*Syzygium tephrodes*	*Syzygium*	Myrtaceae
76	Tcau	*Themeda caudata*	*Themeda*	Poaceae
77	Twal	*Tarennoidea wallichii*	*Tarennoidea*	Rubiaceae
78	Vman	*Vatica mangachapoi*	*Vatica*	Dipterocarpaceae
79	Xhai	*Xanthophyllum hainanense*	*Xanthophyllum*	Xanthophyllaceae

Table S2. a Plant composition of families, genera and species in early succession stage.

	Family	Genus	Species	Genus : Species
1	Euphorbiaceae	5	6	5:6
2	Myrtaceae	4	5	4:5
3	Lauraceae	2	3	2:3
4	Fabaceae	3	3	1:1
5	Guttiferae	2	2	1:1
6	Melastomataceae	2	2	1:1
7	Rubiaceae	2	2	1:1
8	Theaceae	2	2	1:1
9	Annonaceae	1	1	1:1
10	Apocynaceae	1	1	1:1
11	Bignoniaceae	1	1	1:1
12	Capparaceae	1	1	1:1
13	Dilleniaceae	1	1	1:1
14	Dipterocarpaceae	1	1	1:1
15	Ebenaceae	1	1	1:1
16	Flacourtiaceae	1	1	1:1
17	Oleaceae	1	1	1:1
18	Papilionaceae	1	1	1:1
19	Phyllanthaceae	1	1	1:1
20	Polygalaceae	1	1	1:1
21	Rutaceae	1	1	1:1
22	Salicaceae	1	1	1:1
23	Sterculiaceae	1	1	1:1
24	Thymelaeaceae	1	1	1:1
25	Tiliaceae	1	1	1:1
26	Verbenaceae	1	1	1:1

Table S2. b Plant composition of families, genera and species in early-mid succession stage.

	Family	Genus	Species	Genus : Species
1	Ebenaceae	1	3	1:3
2	Myrtaceae	2	4	1:2
3	Moraceae	1	2	1:2
4	Fagaceae	3	5	3:5
5	Euphorbiaceae	6	9	2:3
6	Lauraceae	5	7	5:7
7	Sterculiaceae	3	4	3:4
8	Rubiaceae	5	5	1:1
9	Annonaceae	2	2	1:1
10	Dipterocarpaceae	2	2	1:1
11	Fabaceae	2	2	1:1
12	Flacourtiaceae	2	2	1:1
13	Sapindaceae	2	2	1:1
14	Theaceae	2	2	1:1
15	Bignoniaceae	1	1	1:1
16	Capparaceae	1	1	1:1
17	Connaraceae	1	1	1:1
18	Dilleniaceae	1	1	1:1
19	Guttiferae	1	1	1:1
20	Icacinaceae	1	1	1:1
21	Juglandaceae	1	1	1:1
22	Magnoliaceae	1	1	1:1
23	Melastomataceae	1	1	1:1
24	Meliaceae	1	1	1:1
25	Menispermaceae	1	1	1:1
26	Myrsinaceae	1	1	1:1
27	Oleaceae	1	1	1:1
28	Phyllanthaceae	1	1	1:1
29	Poaceae	1	1	1:1
30	Rosaceae	1	1	1:1
31	Rutaceae	1	1	1:1
32	Santalaceae	1	1	1:1
33	Sapotaceae	1	1	1:1
34	Staphyleaceae	1	1	1:1
35	Styracaceae	1	1	1:1
36	Symplocaceae	1	1	1:1
37	Tiliaceae	1	1	1:1
38	Verbenaceae	1	1	1:1
39	Xanthophyllaceae	1	1	1:1

Table S2. c Plant composition of families, genera and species in mid succession stage.

	Family	Genus	Species	Genus : Species
1	Ebenaceae	1	3	1:3
2	Myrsinaceae	1	3	1:3
3	Oleaceae	1	2	1:2
4	Symplocaceae	1	2	1:2
5	Moraceae	2	3	2:3
6	Sterculiaceae	2	3	2:3
7	Theaceae	2	3	2:3
8	Fagaceae	3	4	3:4
9	Myrtaceae	3	4	3:4
10	Lauraceae	7	8	7:8
11	Euphorbiaceae	6	6	1:1
12	Rubiaceae	5	5	1:1
13	Annonaceae	4	4	1:1
14	Dipterocarpaceae	2	2	1:1
15	Fabaceae	2	2	1:1
16	Sapindaceae	2	2	1:1
17	Sapotaceae	2	2	1:1
18	Apocynaceae	1	1	1:1
19	Aquifoliaceae	1	1	1:1
20	Bignoniaceae	1	1	1:1
21	Burseraceae	1	1	1:1
22	Capparaceae	1	1	1:1
23	Celastraceae	1	1	1:1
24	Connaraceae	1	1	1:1
25	Dilleniaceae	1	1	1:1
26	Flacourtiaceae	1	1	1:1
27	Guttiferae	1	1	1:1
28	Icacinaceae	1	1	1:1
29	Juglandaceae	1	1	1:1
30	Melastomataceae	1	1	1:1
31	Meliaceae	1	1	1:1
32	Poaceae	1	1	1:1
33	Rosaceae	1	1	1:1
34	Rutaceae	1	1	1:1
35	Styracaceae	1	1	1:1
36	Thymelaeaceae	1	1	1:1
37	Verbenaceae	1	1	1:1
38	Xanthophyllaceae	1	1	1:1

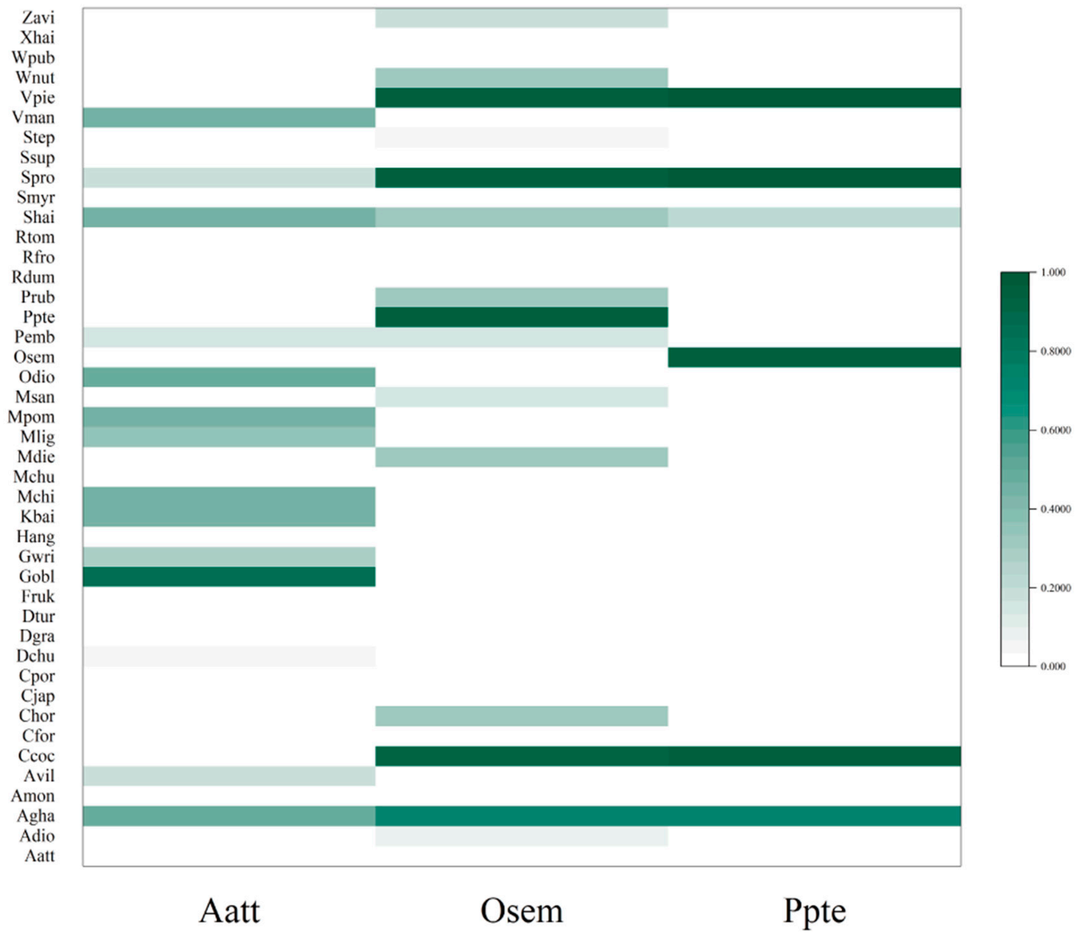

Figure S1. a. Niche overlap of leguminous trees in early successional stages. See TableS1.a for the comparison of plant name abbreviations.

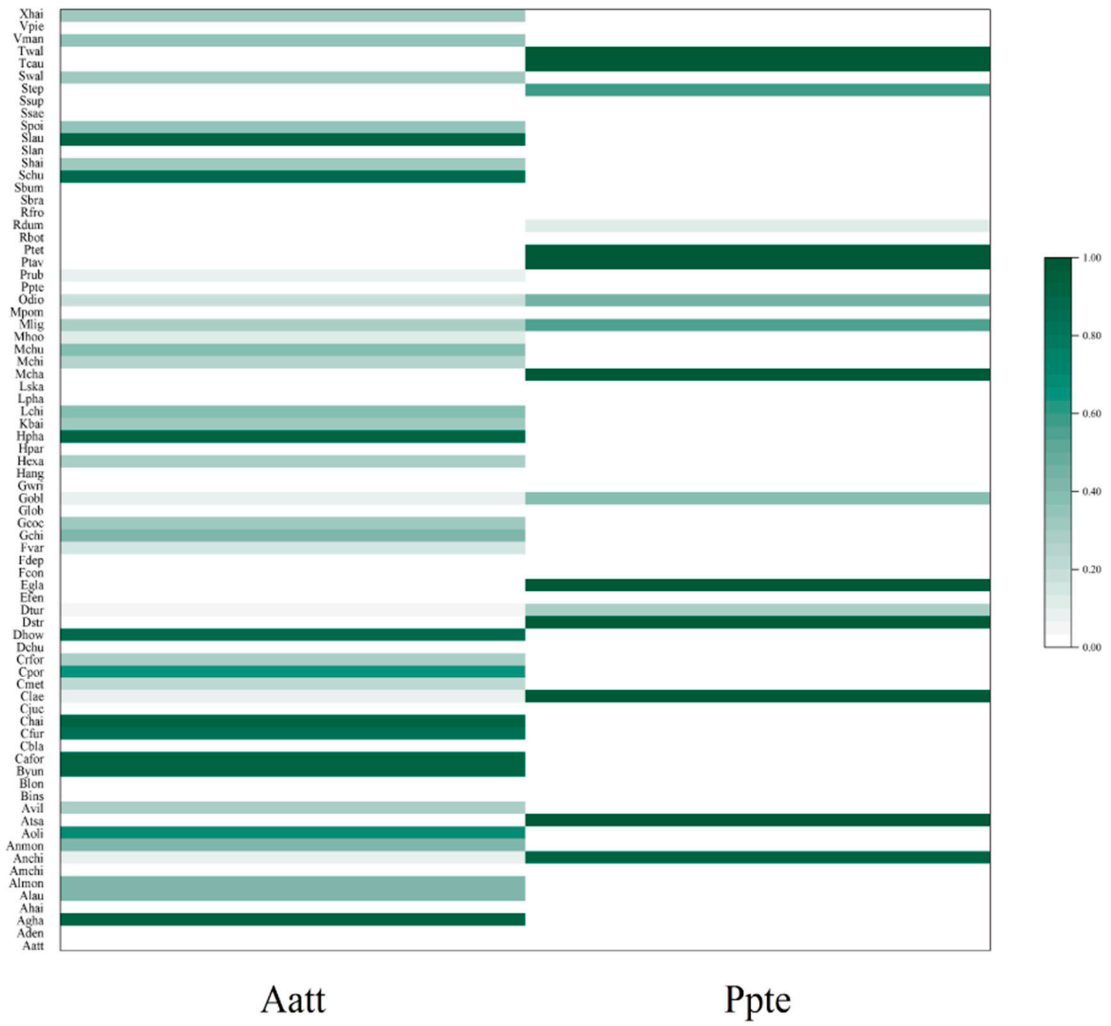

Figure S1. b. Niche overlap of leguminous trees in early-mid successional stages. See TableS1.b for the comparison of plant name abbreviations.

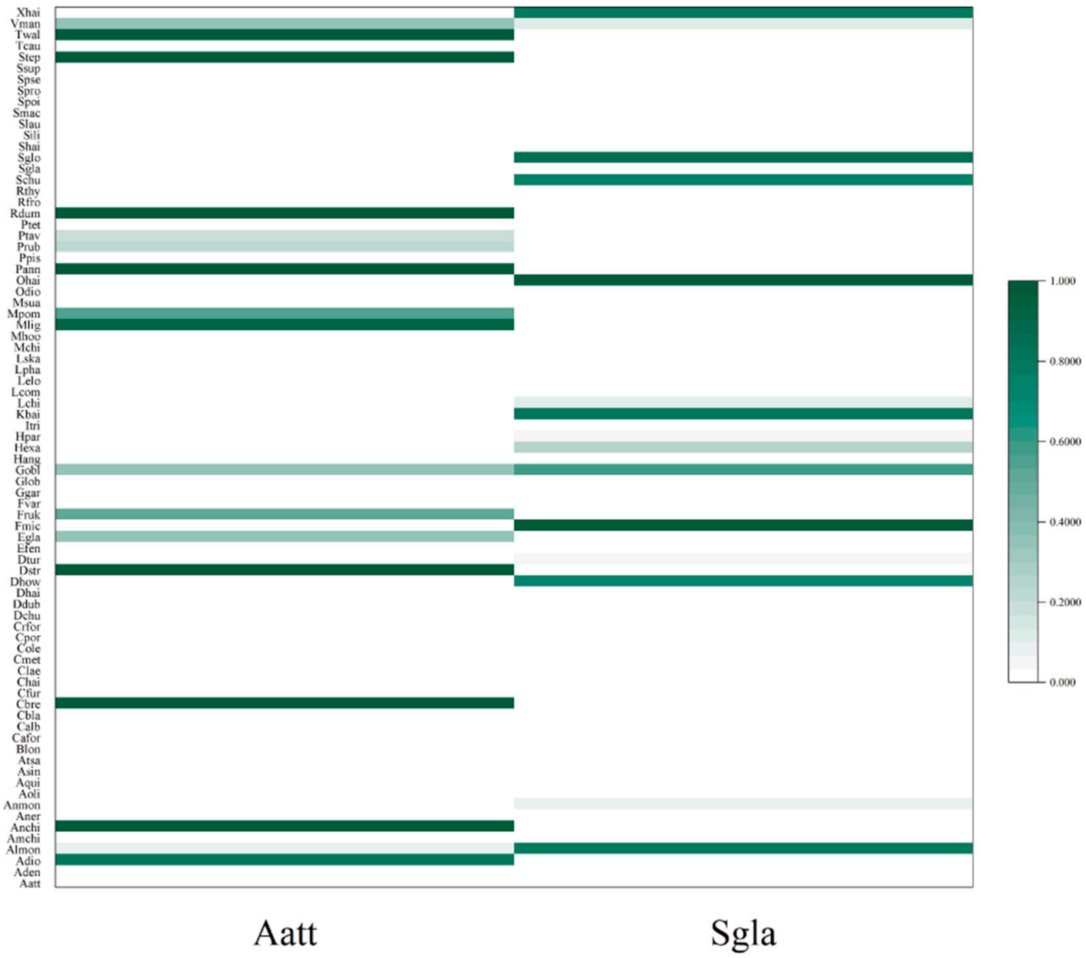

Figure S1. c. Niche overlap of leguminous trees in mid successional stages. See TableS1.c for the comparison of plant name abbreviations.

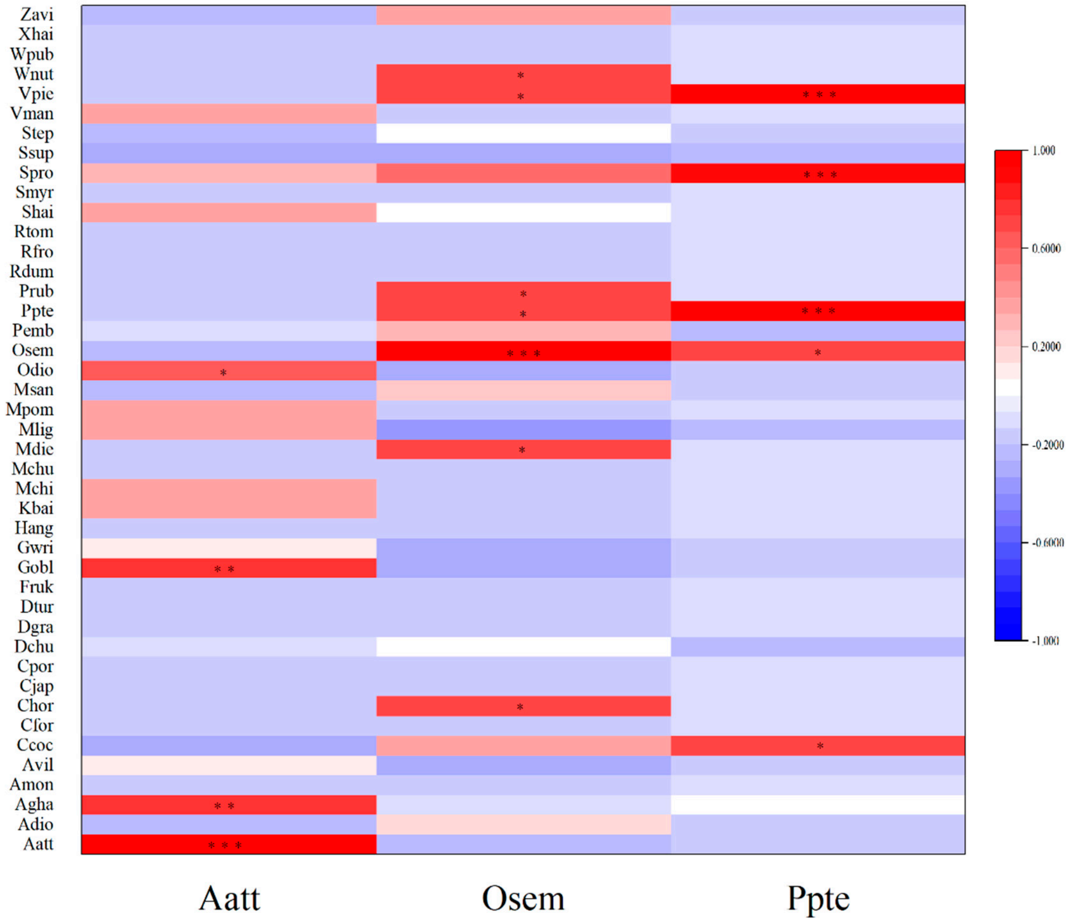

Figure S2. a. Pearson's significance and correlation coefficients of leguminous trees in early successional stages. See TableS1.a for the comparison of plant name abbreviations.

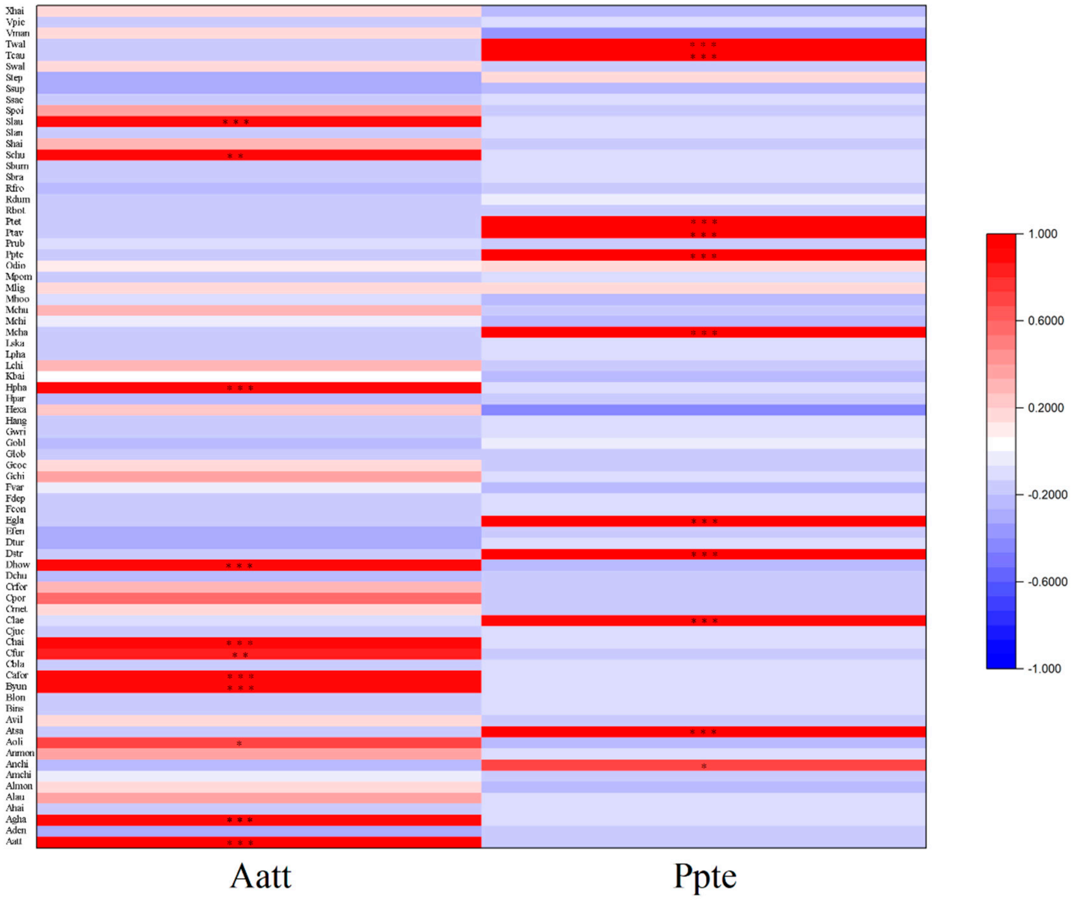

Figure S2. b. Pearson's significance and correlation coefficients of leguminous trees in early-mid successional stages. See TableS1.b for the comparison of plant name abbreviations.

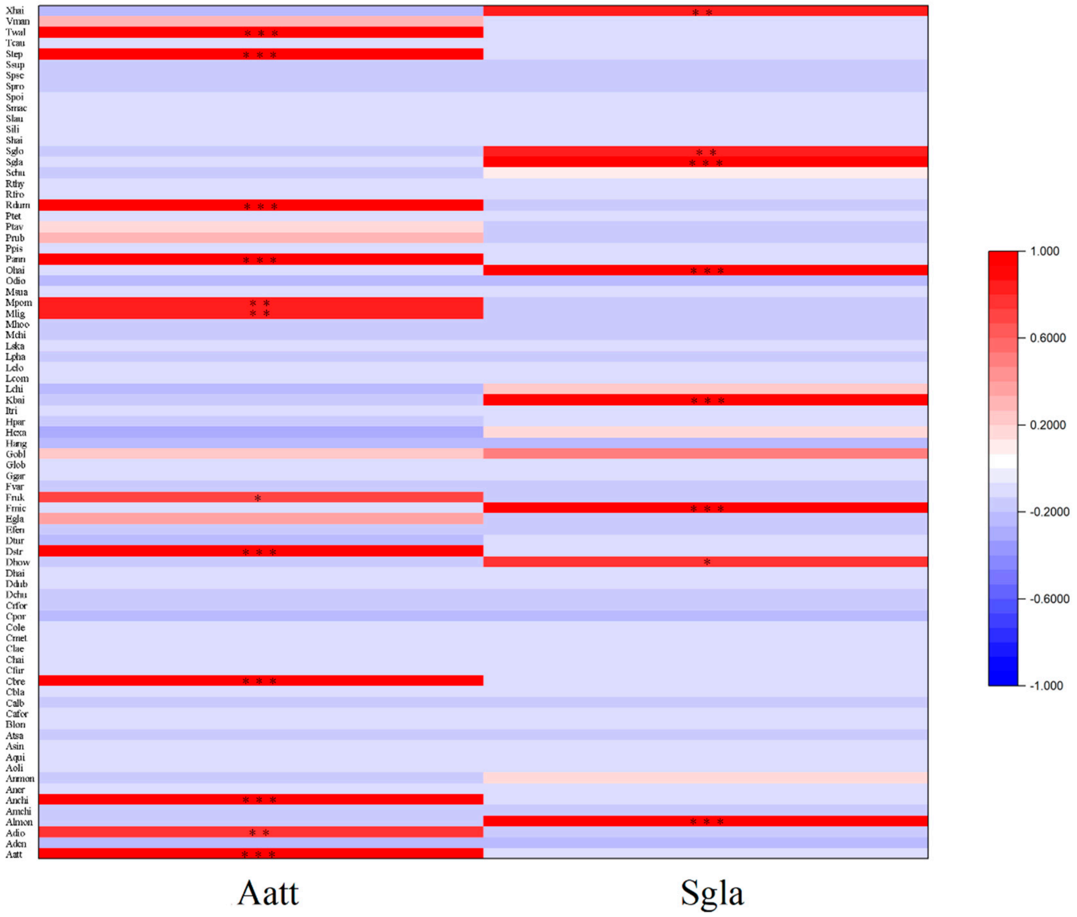

Figure S2. c. Pearson's significance and correlation coefficients of leguminous trees in mid successional stages. See TableS1.c for the comparison of plant name abbreviations.
